# The Role of Cerl2 in the Establishment of Left-Right Asymmetries during Axis Formation and Heart Development

**DOI:** 10.3390/jcdd4040023

**Published:** 2017-12-10

**Authors:** José A. Belo, Sara Marques, José M. Inácio

**Affiliations:** Stem Cells and Development Laboratory, CEDOC, NOVA Medical School, Universidade Nova de Lisboa, 1150-082 Lisboa, Portugal; sara.marques@nms.unl.pt (S.M.); jose.inacio@nms.unl.pt (J.M.I.)

**Keywords:** Cerl2, LR-asymmetry, Nodal signaling, congenital heart diseases

## Abstract

The formation of the asymmetric left-right (LR) body axis is one of the fundamental aspects of vertebrate embryonic development, and one still raising passionate discussions among scientists. Although the conserved role of nodal is unquestionable in this process, several of the details around this signaling cascade are still unanswered. To further understand this mechanism, we have been studying Cerberus-like 2 (Cerl2), an inhibitor of Nodal, and its role in the generation of asymmetries in the early vertebrate embryo. The absence of Cerl2 results in a wide spectrum of malformations commonly known as heterotaxia, which comprises defects in either global organ position (e.g., situs inversus totalis), reversed orientation of at least one organ (e.g., situs ambiguus), and mirror images of usually asymmetric paired organs (e.g., left or right isomerisms of the lungs). Moreover, these laterality defects are frequently associated with congenital heart diseases (e.g., transposition of the great arteries, or atrioventricular septal defects). Here, reviewing the knowledge on the establishment of LR asymmetry in mouse embryos, the emerging conclusion is that as necessary as is the activation of the Nodal signaling cascade, the tight control that Cerl2-mediates on Nodal signaling is equally important, and that generates a further regionalized LR genetic program in the proper time and space.

## 1. The Development and Evolution of Left-Right Asymmetry

Most vertebrates are very active animals, which means they are high-energy consumption organisms. In such demanding environments, the metabolic demand, as well as homeostasis of tissues, had to be guaranteed, and this was attained through the generation of bigger organs [1]. As for smartphones where the limits have to be pushed to package a growing number of features into a small shell, the establishment of a left-right (LR) tissue patterning may have been crucial to accommodate the increasing size of organs, and maintaining or even improving their physiological functions. Indeed, it has been demonstrated that the spirally coiled heart has a higher pumping efficacy than linear tube heart [2]. The outcome of such visceral structure can be translated into the rotation of the gut, the anatomical difference of the lungs, the off-centered stomach, liver, spleen or pancreas. The initial event in this process is cardiogenesis, as the heart is the first organ to be formed during embryo development. It starts to exhibit asymmetry by looping asymmetrically and assuming a leftward tilted position in the thoracic cavity, and it is also the first organ to be affected by LR mispatterning [3].

## 2. Left-Right Establishment

Like any other mechanism involved in the development of the vertebrate embryo, the establishment of LR asymmetry requires a complex interplay of several molecular genetic pathways. Although the understanding of how the LR axis is established has been the focus of several studies that go back to the 18th century, there is not a common model for the process. It is easy to enumerate conserved but also divergent features in LR mechanisms of different animal models [4,5]. However, there are three fundamental steps in the LR embryo patterning that are consensual in all models [4,5,6,7,8,9,10]. First, the initial bilateral symmetry breaking in the left-right organizer (the node in mice, or the Kupffer’s vesicle in zebrafish), a transient structure at the posterior end of the notochord during early somitogenesis. The second step is the propagation of the LR information generated on the node to the mesodermal tissue at the periphery of the embryo, the lateral plate mesoderm (LPM), which will trigger a LR asymmetric cascade of gene expression. The last step is the integration of the LR signaling by the organ primordia and the translation of this information at the cellular level into the asymmetrical growth of tissues, leading to the proper establishment of the organ morphology and position in the body plan.

In the mouse, the hallmark on the establishment of the LR asymmetry is the expression of *Nodal*, a secreted protein member of the transforming growth factor-beta (TGF-β) superfamily on the left-lateral plate mesoderm (L-LPM). It was demonstrated that this expression of *Nodal* on the L-LPM is a consequence, after symmetry breaking, of the upregulation of nodal signals on the left side of the mouse node [11]. 

The mouse node is a temporary structure with a few hundred cells that it positioned between the anterior notochord and the primitive streak in the ventral midline of the embryo during early somitogenesis stages [12]. Although transient, the formation of this LR organizer is very complex, dynamic, and dependent on the activity of several molecules, from transcription factors (e.g., Brachyury) to extracellular matrix proteins (e.g., fibronectin), and signaling pathways (Nodal, Planar Cell Polarity, Notch) [13,14,15,16]. Two types of cells compose the ventral mouse node, the pit cells, which are columnar epithelial cells located in the central region of the ventral node, and the crown cells, squamous epithelial cells located on the edge of the node [17,18,19]. Although both cell types are monociliated, with 9 + 0 architectured cilia projected into the extraembryonic space, the cilia seem to have different functions. Most of the cells in the central pit region possess motile rotating cilia that are located on the posterior side of these dome-shaped cells. The clockwise strokes of these cilia, which tilt towards the posterior side of the cavity, are responsible for the generation of a left-directed extraembryonic fluid flow across the node. The crown cells of the mouse LR organizer have, in 90% of cases, immotile cilia that seem to perceive, integrate, and process the direction and force of the fluid flow. Therefore, it is conceivable that the LR asymmetric cascade of gene expression that governs the further LR structural changes in the embryo starts in the node crown cells as a response to the fluid flow. Because the direction of the fluid seems to be crucial, the leftward fluid flow is, under proper conditions, the trigger for the asymmetric enhancement of *Nodal* expression on the left side of the perinodal region of the ventral node, and subsequent downstream molecular activity of Nodal signaling on the peripheral regions of the embryo. 

## 3. Cerl2 Is Crucial to Tune Nodal’s Bioavailability to the Embryo

**Figure 1:**
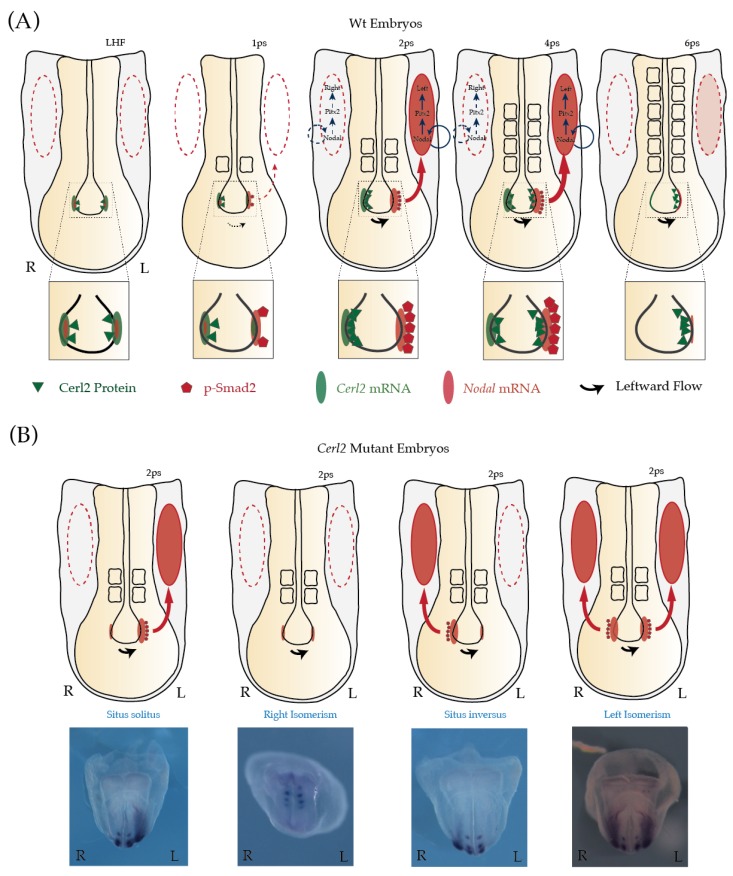
Sequential Nodal activity in left-right asymmetry at the mouse node during early somitogenesis. *Nodal* expression is represented in light red oval, and *Cerl2* expression in light green oval. Cerberus-like 2 (Cerl2) protein is illustrated in green triangles, and the readout of Nodal signaling, pSmad2/3, is indicated in red pentagons. The expression of *Nodal* in the lateral plate mesoderm (LPM) of mouse embryos is represented by the filled red oval. Dashed to thicker lines indicate increase in intensity. (**A**) At the one-somite stage, as soon as the cilia at the node began to rotate generating a weak leftward flow, the asymmetric expression of *Cerl2* (L < R) is established. This local reduction of *Cerl2* mRNA (and Cerl2 protein) in the perinodal cells on the left side of the node results in an increase of active Nodal signal, i.e., pSmad2/3. At the 2-somite stage, Cerl2 protein (green triangles) localizes and prevents the activation of Nodal genetic circuitry on the right side of the embryo (dashed red oval). Later, due to nodal flow, Cerl2 right-to-left translocation shutdowns Nodal activity in the node and consequently affects the activity of Nodal in the LPM (dashed red oval). (**B**) In the absence of Cerl2 in the node, the expression of *Nodal* in LPM becomes randomized.

Cerberus-like 2 (Cerl2)/Dand5, is a secreted 20-kDa protein belonging to the family of TGF-β/Nodal signaling antagonists Cerberus/DAN. Cerl2 is one of the key players in the initial breaking of LR symmetry and on the control of the transmission of LR asymmetry information from the node to the LPM (Figure 1) [20,21]. The significance of Cerl2 in the establishment of LR asymmetry came to light when its expression was first detected in a horseshoe-shaped pattern in the perinodal region of the mouse embryo at early head-fold (EHF) stage [22]. Although resembling the expression of Nodal at this stage, *Cerl2* mRNA assumes a complementary expression pattern to that of *Nodal* by the early somitogenesis stage. Moreover, it was described that the Cerl2-mediated antagonism of nodal signaling requires Cerl2 binding to the ligand Nodal, which consequently prevents the interaction of Nodal with the receptor and subsequent signaling activation [22]. Therefore, at the time, the *Cerl2* expression pattern and its Nodal inhibitory activity, together with the laterality defects observed in the cerl2 KO mutants, placed Cerl2 as a protein at the proper time and place to be involved in LR symmetry breaking. More recently, it has been suggested that *Cerl2* is the earliest gene to become asymmetric in LR gene expression cascade [23]. This asymmetric expression of *Cerl2* (L < R) seems to be established as soon as the cilia at the node began to rotate generating a weak leftward flow. Curiously, in *inv*/*inv* embryos the asymmetric expression of Cerl2 is reversed [24]. Moreover, it was observed that the L < R asymmetry of *Cerl2* is established in the perinodal crown cells, post-transcriptionally, by a robust decay of the *Cerl2* mRNA on the left side via its 3′-UTR [25].

Although still unclear, several lines of evidence suggested that the initial generated fluid flow is sensed via Pkd2, a Ca^2+^-permeable cation channel present in the immotile cilia of the perinodal crown cells, and the subsequent flow-derived signal promotes the degradation or the instability of *Cerl2* mRNA [19]. In addition, it has been also suggested that is the Wnt-β-catenin mediated signaling that regulates the asymmetric degradation of *Cerl2* mRNA [25,26].

As mentioned above, the mouse LR organizer is a small but very a dynamic structure in which each molecule seems to obey a strict script for a correct appearance on stage, both in space and time. That is, the local reduction of *Cerl2* mRNA (and Cerl2 protein) in the perinodal cells on the left side of the node results in an increase of active Nodal signal, that is, in the phosphorylation levels of Smad2/3 (pSmad2/3) at the one-somite stage [21,27]. This asymmetric distribution of Cerl2 protein L < R precedes and causes the asymmetry in pSmad2 distribution, L > R. In addition, Smad2/3 phosphorylation has a symmetrically distributed pattern in Cerl2 knock-out (KO) embryos, highlighting once again the important role of Cerl2 in the induction of pSmad2 asymmetry. 

Curiously, the fluid flow on the node not only produces an asymmetry of the distribution of *Cerl2* mRNA but also of the Cerl2 protein. It was observed in mouse embryos that after its accumulation on the right side of the node, Cerl2 protein displays a nodal flow-dependent right-to-left translocation at the three- to four-somite stages [21]. The effect of the leftward fluid flow on the localization of Cerl2 protein was confirmed by the examination of embryos cultured in the presence of 1% methylcellulose, which mechanically abolishes the ciliary motion and fluid flow. At the five-somite stage, Cerl2 protein localizes preferentially on the left side of the node, exactly when Smad2/3 phosphorylation begins to disappear. Therefore, after preventing any activity of Nodal on the right side of the node, the presence of Cerl2 protein on the left side suppresses the activity of Nodal in the entire node. 

Shortly after an L > R difference in nodal activity in the node is generated, *Nodal* becomes expressed on the left side of the lateral plate mesoderm (L-LPM) [27]. Despite the fact that the presence of Nodal protein in the node is a prerequisite for LPM gene expression, the transport of Nodal, whether directly or not, has never been demonstrated and remains controversial. Nevertheless, several lines of evidence suggest that Nodal might interact with Gdf1, and that sulfated glucosaminoglycans in the extracellular matrix of the intervening tissue are most likely involved in the transport of this complex [28].

**Figure 2:**
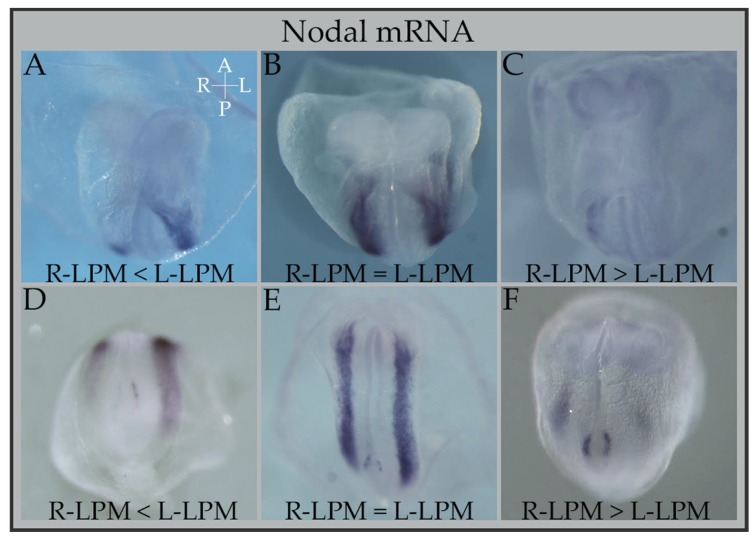
Differential Anterior-posterior bilaterality of Nodal expression in *Cerl2* knock-out (KO) embryos. Bilateral expression of *Nodal* in the lateral plate mesoderm of *Cerl2* KO embryos (50% display bilateral expression: left isomerism) at about E8.0–E8.5 (from 2 to 7 somite pairs). This bilateral expression can be equal in the left or right LPM occupying the totality of both LPMs (panels **B**, **E**); or it can be bilateral only in a more posterior or anterior area (panels **A**, **C**, **D**, **F**). This partial bilaterality can be either more preponderant on the R-LPM (panels **C** and **F**) or on the L-LPM (panels **A**, **D**). Partial bilaterality is set early on as can be seen in panel **F** (2–3 somite embryo), and will last until the end of *Nodal*’s expression in the LPM, at about 7 somites (as can be seen in panel **C**). Embryo axes coordinates are present in panel **A**, in white letters: A: anterior; P: posterior; R: Right; L: Left. R-LPM (right—Lateral Plate Mesoderm); L-LPM (left—Lateral Plate Mesoderm).

The expression of Nodal on the left side of the LPM starts transiently in a small region adjacent to the node (Figure 2), at the 2-somite stage, and then expands along the anterior-posterior (AP) axis, disappearing by the 6-7-somite stage, lasting only several hours. For several years, it was considered that this tight regulation was mediated by the action of *Lefty* (1 and 2) genes on the LPM. These genes, *Lefty 1/2*, are direct targets of Nodal signaling and act as feedback inhibitors by competitive interaction with the Nodal receptors/co-receptors [29,30,31,32]. When an active Nodal signal molecule reaches a certain target region, it will promote the transcription of both *Nodal* and *Lefty 1/2*. Therefore, although Nodal induces its own expression positively, it will also induce the expression of its negative regulators that will eventually terminate the expression Nodal itself. This regulatory relationship between Nodal and Lefty molecules is a well-known type of reaction-diffusion system, where the inhibitor diffuses more rapidly than the activator, and constituting a self-enhancement lateral inhibition (SELI) system [33,34,35]. *Lefty1* is predominantly expressed on the midline or prospective floor plate (PFP) and its expression is also temporary, beginning at the 2-somite stage, near the node, expanding anteriorly, and disappearing by the 5-6-somite stage [31,32,36]. Curiously, an ectopic expression of *Lefty1* was seen on the node of *Cerl2* KO mutants, most probably due to an abnormal number of active Nodal molecules present on the node in the absence of Cerl2 protein [24]. *Lefty2* is expressed primarily in the left LPM restricting the area of Nodal signaling to a particular region in the lateral tissue by the reaction-diffusion patterning mentioned above [29,31,32]. 

However, several observations in mouse embryos support the idea that by regulating the level of active Nodal molecules in the node, Cerl2 regulates the asymmetric pattern of Nodal transmitted to the LPM and correct organ situs program: (1) The absence of Cerl2 in the node results in an upregulation of Nodal signal and the sudden presence of *Lefty1* expression in the node. This ectopic expression of *Lefty1* in the node causes a delay in the onset of Nodal program in the LPM [24]. (2) The local post-transcriptional degradation of *Cerl2* mRNA on the left side of the node, at the two-somite stage, drives the L > R asymmetric distribution of phosphorylated Smad2/3 on the node that is subsequently transduced in the L-LPM. This close correlation between the perinodal distribution of pSmad2/3 and the expression of Nodal in the LPM is evident among several LR mutant mice observed [21,25,27]. (3) Simultaneously, at the two-somite stage, the accumulation of Cerl2 protein on the side of the node prevents the activation of the Nodal cascade on the R-LPM, as predicted by the SELI system model and observed on mouse embryos [21]. (4) The flow-dependent right-to-left translocation of Cerl2 shuts down the Nodal activity on left side of embryo, that is, the presence of Cerl2 protein on the left side of the node by the 6-somite stage terminates the transfer of laterality information to the left side of the embryo and, as a consequence, the expression of *Nodal* in the LPM ceases [21,25,27]. (5) In the absence of both of its inhibitors, in *Cerl2*/*Lefty1* double KO embryos, Nodal activity increases and prolongs its mRNA self-expression not only on the node but also in the LPM. The expression of *Nodal* is bilateral in these mouse mutants, and begins much earlier, as soon as the 1-somite stage, and persists until the 6-somite stage [21,25,27]. 

## 4. Cerl2 and Cardiac Left-Right Development

As mentioned above, the initial LR axis determining event that will result in observable morphological asymmetries is, in vertebrates, the establishment of a left identity which is granted by the left-specific Nodal pathway, first on the node and subsequently transferred to the LPM. The asymmetric activation of this pathway on the left LPM is defined by the expression of *Nodal*, *Lefty2*, and by the transcription factor *Pitx2c* in this lateral zone, with its anterior expression domains reaching into the posterior primary heart field in cells that will be incorporated in the posterior part of primary heart tube later, already with some LR identity cues [3]. 

Alterations in this asymmetric gene expression cascade will cause defects in the left-right development of the organs, and it is known that the worst malformations are associated with symmetrical rather than lateralized arrangement of the organs, this setting being associated with the most complex cases of congenital heart defects (CHD) [37]. For example, the absence of Nodal’s downstream effector gene, *Pitx2c*, will cause gut malrotation, right pulmonary isomerism, heterotaxy, and a spectrum of cardiac defects [38,39,40,41]. Unlike *Nodal*, *Pitx2c* also has cardiac expression; in fact, it is expressed in the heart tube in an asymmetric fashion, maintaining its left side exclusivity of expression, which persists during looping stages until later stages [3]. Interestingly, as reported for Nodal dosage-sensitive and concentration threshold dependent activity, the different levels of expression of Pitx2 are also specific for different developmental processes. As seen by [40] the defects in the *Pitx2* KO can be rescued with hypomorphic *Pitx2c* alleles except for the right atrial isomerism, which requires a higher dosage of *Pitx2c* expression/activity. 

In the case of *Cerl2* mouse mutants, we can observe a spectrum of asymmetry defects defined as situs ambiguous, and a significant mortality rate within a few hours after birth due to cardiac defects [22]. Some of these defects are laterality-associated ones affecting organogenesis and caused by a multitude of LPM expression pattern combinations of *Nodal* and its downstream genes.

In fact, it seems that the absence of Cerl2 antagonism in the node will result in a dosage-dependent randomization of Nodal signaling. Only relying on nodal fluid flow, the expression of *Nodal* in *Cerl2* mutants is seen not only with the correct L-LPM expression pattern, but it is also observed in its inverse (R-LPM-only expression, situs inversus), its absence (right isomerism), or its bilateral expression, namely, both L- and R-LPM. Interestingly, in these last expressions, a certain degree of A-P differential bilaterality of *Nodal* expression is commonly observed. That is, in some embryos bilaterality of LPM expression is seen only near the node, and not more anteriorly or posteriorly, in other cases this bilaterality is seen only more anteriorly in heart-field zones, and sometimes there is a full bilateral LPM expression (left-isomerism) (Figure 2). This reflects the fact that the level of antagonism is required in an exact time window, to regulate the necessary dose of Nodal activity in that time window, and these two variables (time/dosage) will then influence the outcome of the pathway’s establishment significantly. 

The end result in the case of *Cerl2* mutants is a complex combination of phenotypes in which the heart, and/or lungs are not correctly LR specified, but the gut can be looped correctly and stomach correctly placed, or not [22].

The LR-derived organogenesis is accomplished in several ways from the two organ fields that are initially present to either side of the midline, (or straight tubes on top of it) [3]: in the spleen, only the left-side tissue completes differentiation [42], while in the heart tube, it will bend, twist and remodel into its mature form with four chambers that no longer resemble the simple initial tube [43]. For directional looping initiation, several mechanisms have been proposed in different animal models: in zebrafish, it was reported recently that expression of *Prrx1a* in higher levels on the R-LPM, drives through a differential LR epithelial mesenchymal transition (EMT) and cell movements towards the midline, to a leftward displacement of the cardiac posterior pole [44]. 

This asymmetric *Prrx1* expression is, however, dependent on the master gene of the LR determination, *Nodal*. In the mouse, this mechanism is proposed to have been replaced by *Snail1* [44], explaining the heart laterality phenotype observed in these mutants [45]. Taking into account these new findings, it has been found that L-LPM Nodal is necessary to produce correct heart looping, but this process might be controlled by both Nodal-dependent and -independent mechanisms [44,46].

**Figure 3:**
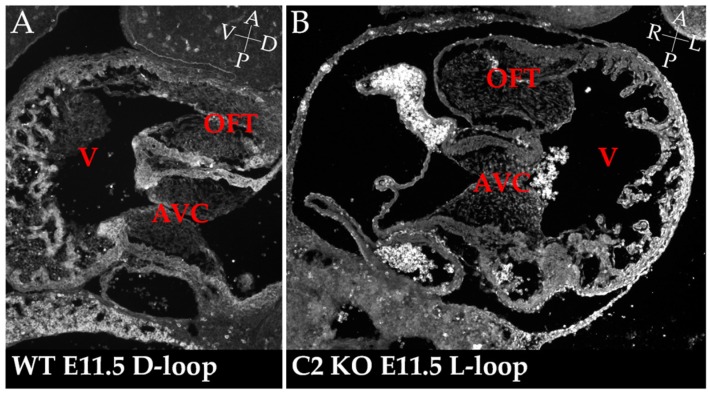
Incorrectly looped hearts in *Cerl2* mutant embryos. Sagittal section of a wild-type (WT) (**A**) and coronal section of *Cerl2* mutant (**B**) hearts after looping stages, at developmental stage E11.5. These sections made in different orientations (sagittal and coronal) show exactly the same anatomical features: the common ventricular chamber (V) and span the outflow tract (OFT) and atrioventricular canal (AVC) cushions of E11.5 mouse embryos. This shows that in the WT there was a correct rightward (D-loop) (**A**), and in the mutant the looping occurred at a different angle, a leftward one (L-Loop) (**B**). Embryo axes coordinates are present in panels **A** and **B**, in white letters: A: anterior; P: posterior; R: Right, L: Left; D: Dorsal; V: Ventral.

In mice and, in particular, in *Cerl2* mutants, which have a defective left-Nodal identity establishment, looping defects are seen in 54% of embryos at E9.5 (L-, and ventral-looped hearts) [22]. It is well known that incorrect looping results in septation defects, and one such example of an incorrectly looped heart is shown in our mutants at a later stage, E11.5, in Figure 3. Atrial septal defects have also been reported in our mouse mutants before [22]. Moreover, in agreement with the mouse results, a variant in the functional domain of DAND5 protein (human Cerl2) has recently been identified in our lab, in patients with both CHD and laterality defects [47].

Cerl2 is not involved in the looping process per se, only indirectly by influencing Nodal LPM pathway program; and unlike *Pitx2c*, *Cerl2* is not expressed in anterior L-LPM or in the primitive heart tube at these stages; however, it will be expressed later on in the heart. In fact, we have uncovered another expression stage for *Cerl2* from E10.5 to E13 [48], although this expression is at a much lower level than the one seen in the node at earlier stages [22].

This later expression time window uncovered a role for Cerl2 during cardiac development that is independent of its function in the early events of LR asymmetry establishment [48]. We propose that the cardiac malformations observed in *Cerl2* null mice result from a combination of both disturbances of laterality and of disruption of the contribution from intrinsic cardiac lineages independent from laterality leading to increased interventricular septum (IVS) and left ventricle (LV) wall thickness. In *Cerl2* mutants, cardiac development proceeds to the 4-chambered heart stage, but in some cases laterality defects such as ventricular septal defects are present.

The more striking cardiac malformation observed in *Cerl2* KO neonates is the massive increase in the left ventricular myocardial wall, with an increase in *Ccnd1* gene expression, pSMAD2 signaling, and *Baf60c/Smarcd3* ratio in embryonic hearts. In accordance with our hypothesis that the overexpression of *Baf60c* could mediate this phenotype, very recently it was reported that in *Baf60c* mutants, embryonic hearts had a thin compact layer, and at E14.5 the ventricular walls were much thinner than WT [49]. Therefore, in addition to its involvement in the first step of asymmetry development, Cerl2 is also required later in cardiac development, independently of its previously characterized role as a negative regulator of the nodal pathway. Several mouse *Cerl2* orthologues have been reported, and those that show close similarities at the protein level are human DAND5 (*p* = 65%), CHARON in zebrafish (*p* = 55%), *Xenopus* COCO (*p* = 53%), and chick CERBERUS (*p* = 51%). All these *Cerl2* orthologues have a conserved essential role in LR axis development [47,50,51,52], moreover, x*Coco* and z*Charon* have been reported to share two important characteristics with m*Cerl2*: they display a right-sided bias of expression that responds to ciliary leftward-driven flow [53,54]. However, so far none of these orthologues have been reported to have an independent role in cardiogenesis.

In summary, for the proper development of the anatomic asymmetries on the visceral organs, and in particular in cardiac development, the intensity and duration of the asymmetric expression of *Nodal-Lefty-Pitx2c* and further genetic programs must be restricted in time and space. Here, we reviewed how, in mice, Cerl2 controls Nodal signaling at the node and the transmission of the LR asymmetry information to the left-lateral plate mesoderm (LPM) in a precise time window, and how this process is crucial for the correct positioning and anatomical development of the heart.
